# Environmental health, clinical studies & biotechnology: new scope for a new era

**DOI:** 10.15252/emmm.202317921

**Published:** 2023-05-17

**Authors:** Kelly M Anderson, Zeljko Durdevic, Jingyi Hou, Lise Roth, Philippe J Sansonetti

**Affiliations:** ^1^ EMBO Heidelberg Germany; ^2^ Institut Pasteur, Collège de France Paris France

## Abstract

EMM Scientific Editors and Editor‐in‐Chief announce that the scope of articles considered will expand, with the goal to enhance our commitment to precision medicine. The scope expansion will cover three major areas: environmental health, clinical studies and case reports, and biomedical technologies

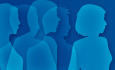

Since its launch in 2009, *EMBO Molecular Medicine* (EMM) has been at the forefront of publishing research at the interface between clinical studies and basic life sciences. Following a discussion with the editorial advisory board, EMM Scientific Editors and Editor‐in‐Chief would like to announce that the scope of articles considered will expand, with the primary goal to enhance our commitment to precision medicine. The EMM scope expansion will accordingly encompass three major areas: environmental health, clinical studies and case reports, and biomedical technologies.

## Environmental health and medicine

In the course of the 20^th^ century, a central public health paradigm switch was marked by a transition in the major causes of mortality from infections and acute malnutrition to chronic conditions such as cardiovascular, metabolic, neurodegenerative diseases, and cancer. The formidable increase in life expectancy, and hence the global aging of the population, has consolidated this transition. In this new paradigm, the health‐disease balance relies on a triad: genetics, lifestyle, and environment. *EMBO Molecular Medicine* has traditionally focused on covering the genetic and molecular basis of these chronic diseases. On occasion, papers would take lifestyle into account, but the emerging field of “environmental health and medicine” remained overall poorly covered. Given the climate and environmental changes upon us, EMM will strongly engage with this emerging field, focusing on molecular aspects. Particular attention will be brought on the crosstalk between the human “exposome” and the host physiology, and its impact on disease development. The scope expansion will also include microbiome sciences, especially those that provide perspective of the human microbiome as a sensor, integrator, and effector of environmental changes. *EMBO Molecular Medicine* will embrace environmental medicine in its functional and mechanistic aspects including exposome studies, toxicology, biomarkers, modeling, and interventions (see, for example, https://www.embopress.org/doi/full/10.15252/emmm.202217014, https://www.embopress.org/doi/full/10.15252/emmm.202013260).

## Human clinical studies and case reports

Since the first modern randomized controlled trials, clinical trials have undergone astonishing progress. Although the evolution of clinical trials has not reached its end point, the COVID‐19 pandemic led to considerable methodological changes and revealed the need for novel adaptive trial designs. In this evolving landscape, EMM will now consider human clinical studies that provide decisive molecular and mechanistic insight into a given disease (epidemiological, pathophysiological, therapeutic, and vaccine studies). *EMBO Molecular Medicine* will also continue welcoming case reports that support hypothesis‐driven research on the disease of investigation (see, for example, https://doi.org/10.15252/emmm.202113936, https://doi.org/10.15252/emmm.201911756, https://doi.org/10.15252/emmm.202012324, https://www.embopress.org/doi/full/10.15252/emmm.202113953).

## Biomedical technologies

Precision medicine (e.g., medicine specifically tailored to an individual patient) cannot be dissociated from molecular medicine. Its development relies on technologies of high molecular resolution for diagnostic and high precision for individualized treatments. In this context, EMM will consider studies that present innovative methods, materials, tools, devices, and technologies with direct translational potential and applicability. This includes, but is not limited to: imaging techniques, drug delivery systems, tissue engineering, and computational/AI‐based technologies.

With this announcement, we encourage authors not to hesitate to contact the EMM editors to discuss the suitability of their manuscript in this new context. As always, we will be delighted to provide advice and assistance before submission just as much as during peer review and revision.

As we implement these changes, we leave you with one strong final message to our readership: *EMBO Molecular Medicine* remains “molecular,” but in a more holistic sense!

